# Molecular cloning and anti-invasive activity of cathepsin L propeptide-like protein from *Calotropis procera* R. Br. against cancer cells

**DOI:** 10.1080/14756366.2018.1444609

**Published:** 2018-03-21

**Authors:** Chang Woo Kwon, Hee Yang, SuBin Yeo, Kyung-Min Park, Ae Jin Jeong, Ki Won Lee, Sang-Kyu Ye, Pahn-Shick Chang

**Affiliations:** a Department of Agricultural Biotechnology, Seoul National University, Seoul, Republic of Korea;; b Department of Pharmacology and Biomedical Sciences, Seoul National University College of Medicine, Seoul, Republic of Korea;; c Center for Food and Bioconvergence, and Research Institute of Agriculture and Life Sciences, Seoul National University, Seoul, Republic of Korea

**Keywords:** *Calotropis procera* R. Br., cysteine protease, cathepsin L, propeptide, cancer cell invasion

## Abstract

Cathepsin L of cancer cells has been shown to play an important role in degradation of extracellular matrix for metastasis. In order to reduce cell invasion, cathepsin L propeptide-like proteins which are classified as the I29 family in the MEROPS peptidase database were characterized from *Calotropis procera* R. Br., rich in cysteine protease. Of 19 candidates, the cloned and expressed recombinant SnuCalCp03-propeptide (rSnuCalCp03-propeptide) showed a low nanomolar *K*_i_ value of 2.3 ± 0.2 nM against cathepsin L. A significant inhibition of tumor cell invasion was observed with H1975, HT29, MDA-BM-231, PANC1, and PC3 with a 76, 67, 67, 63, and 79% reduction, respectively, in invasion observed in the presence of 400 nM of the rSnuCalCp03-propeptide. In addition, thermal and pH study showed rSnuCalCp03-propeptide consisting of secondary structures was stable at a broad range of temperatures (30–70 °C) and pH (2–10, except for 5 which is close to the isoelectric point of 5.2).

## 1. Introduction

Cysteine proteases of the papain superfamily have been implicated in a number of invasive processes. Especially, cathepsin L is upregulated in a variety of malignancies: breast, lung, gastric, colon, head and neck carcinomas, melanomas, and gliomas^1–5^. In addition, the level of cathepsin L expression correlates positively with the degree of malignancy in metastatic tumor development because cell detachment can be partly explained by cathepsin L-mediated cleavage of E-cadherin^6^. Therefore, cathepsin L constitutes an attractive target for the development of inhibitors as promising therapeutic agents.

A variety of compounds are known as inhibitors that can repress cysteine protease activity. These inhibitors have been developed based on the interaction between the active site residue and inhibitor and made use of the known substrate specificities of the enzymes. Inhibition selectivity for the cysteine proteases as opposed to other classes of proteases could be achieved in this manner^7–9^. High-molecular weight protein inhibitors show higher enzyme selectivity than chemical inhibitors binding cysteine residues at the active site due to the stereospecificity of the protein-protein interaction. Therefore, utilization of an inhibitory domain (propeptide) in cysteine protease zymogen is useful to obtain natural protein inhibitors with high inhibitory efficiency.

A number of studies have indicated that propeptide released from the autocatalytic activation of cysteine cathepsin zymogen is a potent inhibitor of their cognate enzymes *in vitro*^10^. The propeptide chain folds on the surface of the enzyme in an extended conformation and runs through the active-site cleft, in the opposite direction to the substrate, thereby blocking access of the latter to the active site, which is already formed in the zymogen^11^. However, little is known about the enzyme selectivity among the plant-derived propeptide^12^.

A few studies have been accomplished on the cysteine proteases in *Calotropis procera* R. Br.^13–16^. Recently, we reported that *Calotropis procera* R. Br. can express at least 20 cysteine proteases, which are highly homologous to the cathepsin L^17^. As the amino acid sequences of the propeptide proteins are homologous to the prodomains of cathepsin L, these inhibitors have been classified as cathepsin L propeptide-like inhibitors. Comprehensive details are available in the MEROPS peptidase database under family I29 and the propeptide of the cathepsin L subfamily (cathepsins L, V, K, S, W, F, and H) contains a 100-residue propeptide with two conserved motifs: a highly conserved ERFNIN and the GNFD motif.

Therefore, we selected and characterized the potent propeptide candidates from *Calotropis procera* R. Br., which is rich in cysteine protease, through comparative structural analysis based on the amino acid sequence and inhibitory activity against human cathepsin L. Furthermore, the medicinal properties of the propeptide were investigated in a series of *in vitro* tumor cell invasion assays.

## 2. Materials and methods

### 2.1. Chemicals

L-*trans*-Epoxysuccinyl-leucylamido(4-guanidino)-butane (E-64), dithiothreitol (DTT), Dimethyl sulfoxide (DMSO), benzyloxycarbonyl-L-phenylalanyl-L-arginine-7-amido-4-methylcoumarin (Z-Phe-Arg-AMC), isopropyl β-D-thiogalactoside (IPTG), antibiotics, and amino acids were purchased from Sigma-Aldrich (St Louis, MO). Active cathepsin L, human recombinant protease, was purchased from Biovision (Milpitas, CA). Dulbecco's modified Eagle's medium (DMEM) and fetal bovine serum (FBS) were purchased from Cambrex (Walkersville, MD).

### 2.2. Cloning of recombinant cathepsin L propeptide-like protein

Cysteine protease unigenes from *Calotropis procera* R. Br. were translated over six frames by the ExPASy translate tool and protein functional domains were predicted by InterProScan 4 web program^17^. The three-dimensional structure models of propeptide domains were generated by the SWISS-MODEL homology-modelling. The complementary DNA (cDNA) encoding the propeptide of cysteine cathepsin L was PCR cloned using a cDNA library of *Calotropis procera* R. Br. The primers, SnuCalCp forward and SnuCalCp reverse, were used to amplify the propeptides constructed from cDNA sequencing (RNA-Seq) data and the restriction sites (Nde1 and Xho1) were employed respectively (Supplementary Table S1). All of the prodomains were cloned into the Nde1 and Xho1 site of the pET29b(+) expression vector incorporating a C-terminal hexahistidine tag for downstream purification and were transformed in *Escherichia coli* DH5α cells. Recombinant plasmids were verified and transformed in *Escherichia coli* BL21(DE3) star cells.

### 2.3. Expression and purification of recombinant cathepsin L propeptide-like protein

Expression of the propeptide was initiated by a 1:10 (v/v) dilution from an overnight subculture into Luria-Bertani media. The diluted culture broth was shaken at 37 °C until the optical density at 600 nm reached 0.5–0.7 and recombinant propeptide was induced by the addition of IPTG to a final concentration of 0.5 mM at 20 °C. After 20 h induction, cells were collected by centrifugation at 10,000× g for 10 min. The cell pellet was resuspended in 50 mM trisaminomethane (Tris)/hydrochloride (HCl) pH 8.5, 300 mM sodium chloride (NaCl), and 10 mM imidazole and were lysed by sonication. The lysate was centrifuged at 12,000×g for 20 min and the supernatant was collected. The supernatant was applied to an immobilised metal affinity chromatography column charged with nickel (Ni^2+^) ions. Column-bound component was washed with a further five column volumes of 50 mM Tris/HCl pH 8.5, 300 mM NaCl, and 20 mM imidazole and then eluted with 50 mM Tris/HCl pH 8.5, 300 mM NaCl, and 250 mM imidazole. The purified recombinant propeptide was analyzed using size exclusion chromatography and sodium dodecyl sulfate polyacrylamide gel electrophoresis (SDS-PAGE).

### 2.4. Cathepsin L inhibition assay

Recombinant propeptide protein samples of various concentrations in 20 µL were mixed with 0.8 ml cathepsin L solution (final concentration: 0.2 nM cathepsin L/100 mM sodium phosphate/10 mM EDTA, pH 6.0) and the mixture was incubated at 30 °C for 5 min. Then, 0.1 ml of 1.2 µM Z-Phe-Arg-AMC (from a 1 mM stock solution in DMSO) for slow-binding inhibition ([*S*] ≪ *K*_m_, for this substrate *K*_m_ was estimated to be 1.92 µM under the experimental conditions) was added to start the reaction. The activity was analysed by the liberation of 7-amino-4-methylcoumarin (AMC: excitation wavelength = 355 nm and emission wavelength = 460 nm) from the synthetic peptide of Z-Phe-Arg-AMC as a substrate with an automated microtiter plate spectrofluorometer.

Under the experimental conditions used, progress curves for the inhibition of cathepsin L by recombinant propeptide at pH 6.0 followed typical slow-binding kinetics as defined by the equation:

$$[P]=v_{i}*t+\frac{\left(v_{i}-v_{s})[1-\exp^{\left(-k_{obs}*t\right)}\right]}{k_{\text{obs}}}$$

where *P* is the product formed, *v_i_* and *v*_s_ are the initial and steady-state velocities, respectively, *t* is the reaction time, and *k*_obs_ is the rate constant for inhibition. Nonlinear regression using the program SigmaPlot 12.0 (Systat Software Inc., San Jose, CA) provided the individual parameters (*v*_i_, *v*_s_, and *k*_obs_) for each progress curve.

### 2.5. Active-site titration of papain and cathepsin L with E-64

Active-site titration was performed as described by Barrett et al.^18^. Working solutions of the irreversible cysteine protease inhibitor (E-64) were prepared from a 1.0 mM stock solution. E-64 solutions of various concentrations were added to the enzyme solution instead of recombinant propeptide and the mixture was preincubated for 5 min at 40 °C. The residual activity of the enzyme was determined by adding a substrate.

### 2.6. Reverse transcription polymerase chain reaction (PCR)

RNA was extracted from the MDA-MB-231 epithelial, human breast cell line using an RNeasy mini kit (Qiagen, Venlo, Netherlands‎). First-strand cDNA was synthesized from 1 µg of total RNA using 1 µL of Quantiscript Reverse Transcriptase (Qiagen) according to the manufacturer’s protocol. PCR amplification was conducted using the following conditions: 40 cycles of 95 °C for 1 min, 55 °C for 90 s, and 72 °C for 1 min followed by a final incubation at 72 °C for 5 min. Primer sequences for target genes were as follows: cathepsin S forward GGGTACCTCATGTGACAAG and reverse TCACTTCTTCACTGGTCATG, cathepsin L forward ATGAATCCTACACTCATCCTTGC and reverse TCACACAGTGGGGTAGCTGGCTGCTG, cathepsin K forward ATGTGGGGGCTCAAGGTTCTGC and reverse TCACATCTTGGGGAAGCTGGCC, cathepsin V forward ATGAATCTTTCGCTCGTCCTGGC and reverse TCACACATTGGGGTAGCTGGC, and actin forward ATCTGGCACCACACCTTCTACAATGAGCTGCG and reverse CGTCATACTCCTGCTTGCTG ATCCACATCTGC.

### 2.7. 3-[4,5-diethylthiazol-2-yl]-2,5-diphenyltetrazolium bromide (MTT) cell viability assay

Cell viability was evaluated by an MTT assay, which measures the mitochondrial reduction of MTT to formazan. MDA-MB-231 cells were seeded in a 96-well plate at a density of 5 × 10^4^ cells per well and were then incubated at 37 °C and 5% carbon -di-oxide (CO_2_) until confluence. Then, one group of cells was treated with 10% FBS DMEM as a control and the other groups of cells were treated with 10% FBS DMEM supplemented with 25, 50, 100, 200, 300, 400, 500, and 600 nM recombinant SnuCalCp03-propeptide (rSnuCalCp03-propeptide).

### 2.8. *In vitro* cell invasion assay

H1975, HT29, MDA-BM-231, PANC1, and PC3 invasion assays were carried out using 5 × 10^5^ cells/Transwell chamber (8 µm, Corning Costar Co., Cambridge, MA). The lower and upper parts of the Transwell were coated with 10 µL of type I collagen (0.5 mg/mL) and 20 µL of 1:2 (v/v) mixture of Matrigel:DMEM, respectively. Cells were plated on the Matrigel-coated Transwell in the presence of rSnuCalCp03-propeptide with predetermined concentrations. The medium in the lower chambers also contained 0.1 mg/mL FBS. The inserts were incubated for 24 h at 37 °C and 5% CO_2_. Cells that had not invaded were removed and cells that invaded to the lower surface of the membrane were fixed with methanol and stained with 1% crystal violet. Random fields were counted under a light microscope. The results were expressed using untreated control cells (0 nM) as 100% cell invasion and all other readings were expressed as average percentage ± standard deviation cell invasion.

### 2.9. Cathepsin L-like subfamily activity

The level of cathepsin L-like subfamily activity from MDA-MB-231 was determined by fluorometric assay. Conditioned medium was retained and cell lysates were prepared using a 100 mM potassium phosphate (pH 6.0) lysis buffer containing 100 mM NaCl and 0.1% triton X-100. The levels of cathepsin L-like activity were analysed by incubation sample (200 µg/mL). The substrate of Z-Phe-Arg-AMC (5 µM) was added and the rate of substrate hydrolysis at 30 °C was monitored every 1 min over a period of 60 min.

### 2.10. Thermal stability of recombinant propeptide

The thermal stability of propeptide (1 µg/µL) was elucidated by incubation of protein at different temperatures (30, 40, 50, 60, 70, 80, 90, and 100 °C) for 30 min. After the samples were cooled at 4 °C for 10 min, cathepsin L inhibition assays were performed. The stability in a wide range of pH was also determined. Samples of propeptide (1 µg/µL) were prepared in 100 mM glycine-HCl buffer (pH 2–3), 100 mM sodium acetate-acetic acid buffer (pH 4–5), 100 mM sodium phosphate buffer (pH 6–7), 100 mM Tris-HCl buffer (pH 8.0), and 100 mM glycine-HCl buffer (pH 9–10). After incubation in each buffer for 24 h at 4 °C, the samples were dialyzed against 100 mM sodium phosphate buffer (pH 6.0) and the inhibitory activity toward cathepsin L was analysed using Z-Phe-Arg-AMC as a substrate.

### 2.11. Circular dichroism (CD)

Far-ultraviolet (190–260 nm) CD spectra were obtained from the CD spectrometer (Chirascan^TM^-plus, Applied Photophysics, Ltd., Leatherhead, Surrey, UK) and temperature-regulated cells (25 °C) with 0.5 mm path and 1.0 nm bandwidth. The spectra were obtained at pH 4–6 in the media mentioned above.

### 2.12. Statistical analysis

The Student’s *t*-test was used to determine the statistical significance of the reduction in invasion observed in the presence of the rSnuCalCp03-propeptide.

## 3. Results

### 3.1. Expression of the rSnuCalCp-propeptide

The eight propeptide domain sequences of SnuCalCp02, SnuCalCp03, SnuCalCp08, SnuCalCp12, SnuCalCp14, SnuCalCp15, SnuCalCp16, and SnuCalCp17 showed high similarity with the cathepsin L propeptide sequence (>40 identity) and were selected as candidates for inhibitor of cathepsin L (Table 1). In order to generate rSnuCalCp-propeptide, the DNA sequences encoding the prodomain were amplified and cloned into the bacterial expression vector, enabling the expression of the domain with a COOH-terminal (His)_6_ tag at the expected molecular weight of 13–16 kDa. Expression for the propeptides of SnuCalCp02, SnuCalCp03, SnuCalCp12, SnuCalCp15, and SnuCalCp16 from BL21(DE3) star *Escherichia coli* cells could be inducible and regulated by the addition of IPTG, as demonstrated by analysis of cell lysates using SDS-PAGE and Coomassie blue staining (Supplementary Figure S1(A)). Cells were harvested and lysed and the soluble (His)_6_-tagged proteins were loaded onto a nickel-charged nitrilotriacetic acid column. The column-bound proteins were eluted using imidazole (250 mM) and were subsequently loaded onto a Hitrap Q ion exchange column and eluted using a NaCl gradient (0–400 mM). Purified rSnuCalCp03-propeptide could be identified by the inhibitory assay of eluted fractions from the purification peak by size exclusion chromatography (Supplementary Figure S1(B)). All rSnuCalCp propeptides could be expressed and purified using the same methods.

**Table 1.** Structural comparison of SnuCalCp propeptide-like proteins with papain-like cysteine proteases.

Unigene ID	PDB ID	Molecule (Species)	Identity	Resolution^a^
SnuCalCp01	4qrx.2.A	Pro-papain (*Carica papaya*)	58%	3.1 Å
SnuCalCp02	3f75.1.B	Cathepsin L propeptide (*Toxoplasma gondii*)	42%	2.0 Å
SnuCalCp03	2o6x.1.A	Secreted cathepsin L1 (*Fasciola hepatica*)	44%	1.4 Å
SnuCalCp04	3f75.1.B	Cathepsin L propeptide (*Toxoplasma gondii*)	37%	2.0 Å
SnuCalCp05	3f75.1.B	Cathepsin L propeptide (*Toxoplasma gondii*)	39%	2.0 Å
SnuCalCp07	3qj3.1.A	Cathepsin L propeptide (*Tenebrio molitor*)	32%	1.8 Å
SnuCalCp08	3f75.1.B	Cathepsin L propeptide (*Toxoplasma gondii*)	46%	2.0 Å
SnuCalCp09	4qrx.2.A	Pro-papain (*Carica papaya*)	67%	3.1 Å
SnuCalCp10	4qrx.1.A	Pro-papain (*Carica papaya*)	58%	3.1 Å
SnuCalCp11	4qrx.1.A	Pro-papain (*Carica papaya*)	65%	3.1 Å
SnuCalCp12	2o6x.1.A	Secreted cathepsin L1 (*Fasciola hepatica*)	44%	1.4 Å
SnuCalCp13	3qt4.1.A	Cathepsin L-like midgut cysteine proteinase (*Tenebrio molitor*)	32%	2.1 Å
SnuCalCp14	3f75.1.B	Cathepsin L propeptide (*Toxoplasma gondii*)	44%	2.0 Å
SnuCalCp15	2o6x.1.A	Secreted cathepsin L1 (*Fasciola hepatica*)	41%	1.4 Å
SnuCalCp16	3f75.1.B	Cathepsin L propeptide (*Toxoplasma gondii*)	44%	2.0 Å
SnuCalCp17	3f75.1.B	Cathepsin L propeptide (*Toxoplasma gondii*)	40%	2.0 Å
SnuCalCp18	4qrx.1.A	Pro-papain (*Carica papaya*)	38%	3.1 Å
SnuCalCp19	4qrx.1.A	Pro-papain (*Carica papaya*)	50%	3.1 Å
SnuCalCp20	4qrx.1.A	Pro-papain (*Carica papaya*)	49%	3.1 Å

^a^The resolution was obtained from X-ray crystal diffraction.

### 3.2. Enzyme inhibition assay

**Figure 1.**
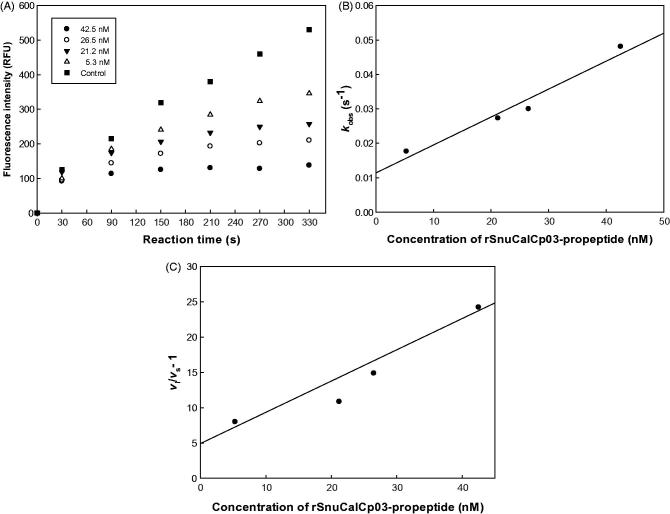
The inhibitory activity of rSnuCalCp03-propeptide against cathepsin L. (A) Protease activity was assessed by monitoring cleavage of fluorogenic substrate, Z-F-R-AMC, in the presence of increasing concentration of rSnuCalCp03-propeptide. (B) Replot of the observed rate constant (*k*_obs_) from the inhibition of mature cathepsin L by rSnuCalCp03-propeptide. (C) Plot of (*v*_i_/*v*_s_ − 1) versus the concentration of rSnuCalCp03-propeptide.

The inhibitory activity of expressed rSnuCalCp-propeptide was analysed initially against recombinant mature human cathepsin L, employing a steady-state fluorometric assay. To evaluate the inhibitory ability of the five expressed recombinant propeptides, the half maximal inhibitory concentration (IC_50_) was determined. Of the five rSnuCalCp-propeptides, rSnuCalCp03-propeptide exhibited the lowest IC_50_ value of 19 ± 2.4 nM during the cathepsin L-catalyzed hydrolysis of Z-Phe-Arg-AMC (Table 2. Therefore, we selected rSnuCalCp03-propeptide for subsequent experiments. The typical time course of cathepsin L-catalyzed hydrolysis in the presence of various concentrations of the rSnuCalCp03-propeptide is shown in Figure 1(A). This time course is indicative of the action of a slow-binding reversible inhibitor.

**Table 2.** Inhibitory ability of five expressed recombinant propeptides.

Expressed recombinant propeptide	IC_50_ (nM)
rSnuCalCp02-propeptide	150 ± 2.6
rSnuCalCp03-propeptide	19 ± 2.4
rSnuCalCp12-propeptide	140 ± 3.2
rSnuCalCp15-propeptide	30 ± 2.2
rSnuCalCp16-propeptide	100 ± 6.2

### 3.3. MTT cell viability assay

To confirm that the observed reduction in tumor invasion was not simply due to antiproliferative or cytotoxic effects of the rSnuCalCp03-propeptide, MTT assays were performed (Supplementary Figure S2). MDA-MB-231 cells were incubated with the rSnuCalCp-propeptide (25, 50, 100, 200, 300, 400, 500, and 600 nM) for 24 h and the number of viable cells was compared with each treatment. No significant effect on cell viability by the rSnuCalCp03-propeptide or control protein was detected. Therefore, the anti-invasive effect observed was solely due to the inhibition of the cathepsin L-like protease, which facilitates tumor invasion.

### 3.4. Inhibition of tumor cell invasion by rSnuCalCp03-propeptide

The presence of mRNA for each protease was clearly evident in H1975, HT29, MDA-MB-231, PANC1, and PC3 cell line, with the exception of cathepsin K (Figure 2(A)), for which the expression was almost negligible in the MDA-MB-231 breast carcinoma cell line. In addition, the activity of the cathepsin L-like proteases was determined by fluorometric assay in the conditioned medium and cell lysate, further showing that these proteases are expressed in and secreted by these tumor cell lines (Figure 2(B)). The rSnuCalCp03-propeptide was found to attenuate the rate of tumor cell invasion (Figure 2(C–E)). Inhibitory extents exerted by the rSnuCalCp03-propeptide of 400 mM were 76, 67, 67, 63, and 79% against H1975, HT29, MDA-MB-231, PANC1, and PC3, respectively.

Figure 2.*In vitro* cell invasion assay. (A) The relative expression levels of the cathepsin L-like protease in MDA-MB-231 cells. Amplification of the β-actin was used as an internal control. (B) The relative activity levels in MDA-MB-231 cell lysate and media. (C) The inhibition effect of the rSnuCalCp03-propeptide on H1975, HT29, MDA-MB-231, PANC1, and PC3 cell invasion. The photograph of invaded MDA-MB-231 cells to lower part of Transwell coated with type I collagen. Incubated without (D) and with (E) rSnuCalCp03-propeptide. The Student’s *t* test was used to determine the statistical significance of the reduction in invasion observed in the presence of the rSnuCalCp03-propeptide (**p < .01).

**Figure:**
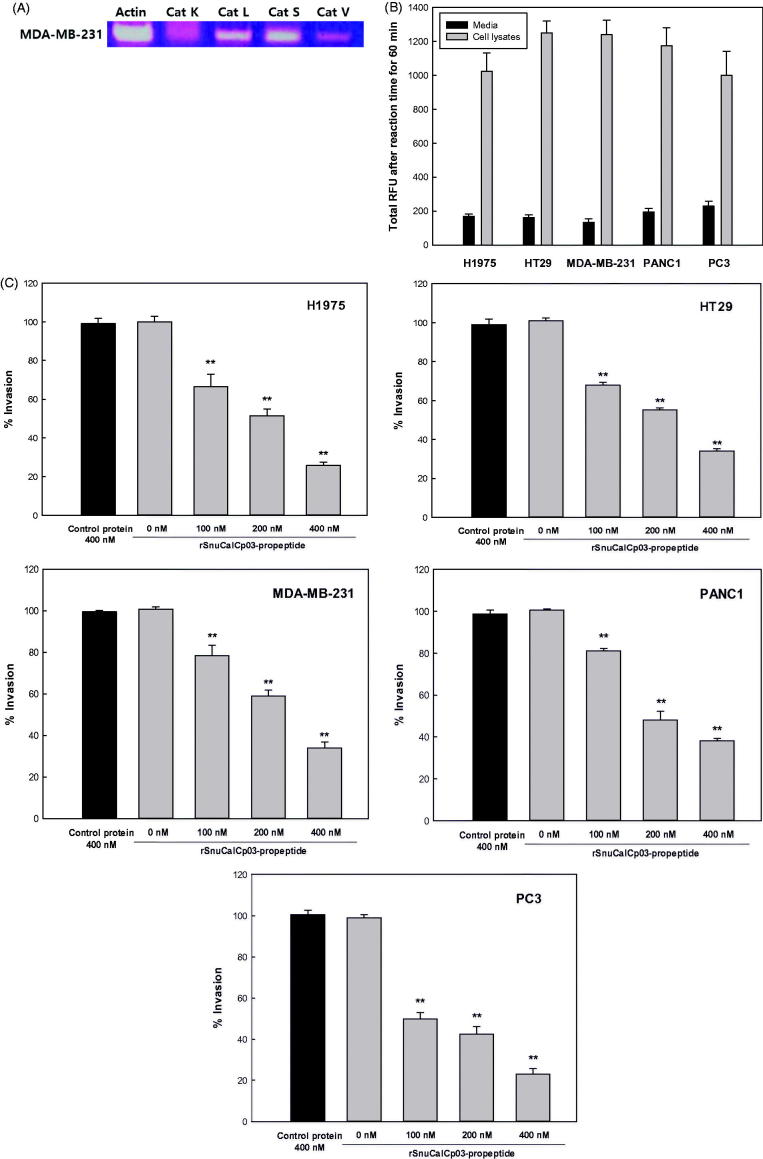

**Figure:**
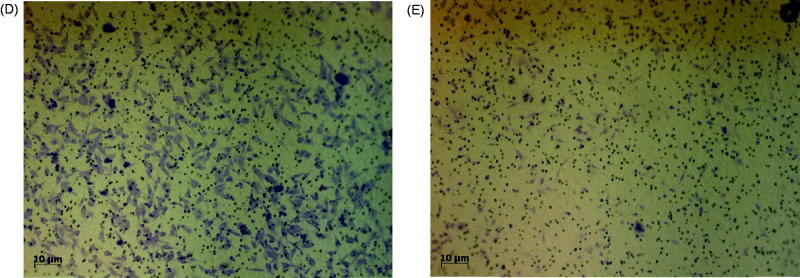

### 3.5. Thermal and pH stability

**Figure 3.**
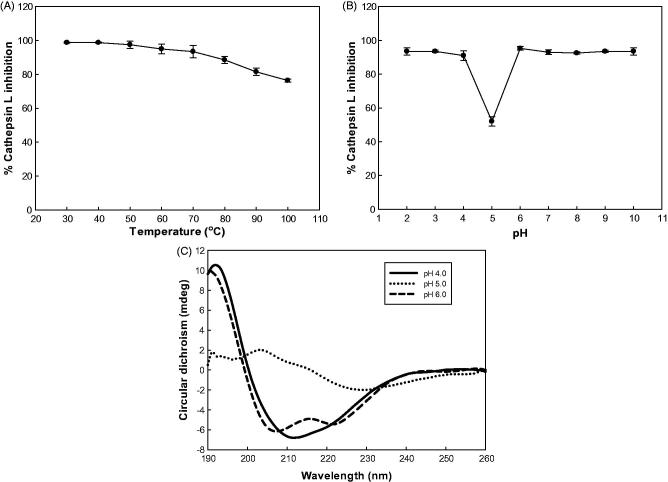
Effect of temperature and pH on inhibitory activity and secondary structure of rSnuCalCp03-propeptide. (A) Temperature stability profile of the rSnuCalCp03-propeptide against cathepsin L enzyme activity. (B) pH stability profile of the rSnuCalCp03-propeptide against cathepsin L enzyme activity. (C) Far UV CD spectra of rSnuCalCp03-propeptide at different pH values.

The study of the temperature effect on rSnuCalCp03-propeptide showed that the inhibitory activity was stable at the temperature below 70 °C and at pH 2,3, and from 6–10 (Figure 3(A,B)). The inhibitory activity was dramatically decreased at pH 5which is close to theoretical isoelectric point of 5.2.

### 3.6. pH-dependent conformational changes of the propeptide

At pH 6, an intense positive band at 193 nm, an intense negative band at 208 nm, and a shoulder at 218 nm suggest a high proportion of amino acid residues integrated in α-helical secondary structures. The CD spectrum at pH 5 showed a dramatic difference in the intensities of the two bands. At pH 4, there was no shoulder at 218 nm because the secondary structure alteration was induced by acidification.

## 4. Discussion

**Figure 4.**
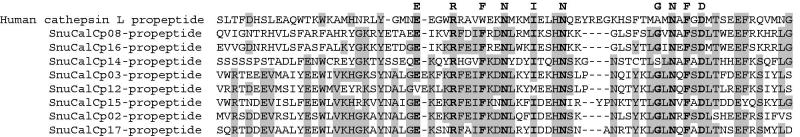
Multiple alignment analysis of deduced amino acid sequences of SnuCalCp-propeptides with human cathepsin L propeptide. Identical and conserved amino acid residues are darkly shaded and conserved signatures (ERFNIN and GNFD) are highlighted in bold.

*Calotropis procera* R. Br., a traditional medicinal plant in India, is a promising source of cysteine proteases and several proteases such as Procerain, Procerain B, CpCp-1–3 have been isolated and characterised^13–15^. Also, we have previously reported the cDNA sequences encoding the full open reading frame of these cysteine proteases from *de novo* transcriptome assembly using Trinitiy and Velvet-Oases^17^. Interestingly, we obtained 20 different cysteine protease gene sequences consisting of a signal sequence domain, I29 inhibitor domain, and peptidase C1A domain. These proteases contain the highly conserved ERFNIN motif, which is present in the long α-helix that is a major part of the cathepsin L-like propeptide scaffold. Another highly conserved GNFD motif is found in this family. We selected and characterized the potent propeptide candidates through comparative structural analysis based on the amino acid sequence and inhibitory activity against human cathepsin L. As shown in Figure 4, the detection of the ERFNIN-GNFD motif in the propeptide of cysteine proteases clearly implies a relationship to the cathepsin L group and inhibition ability of the proteolytic activity against cathepsin L. Of eight potential candidates, the cloned and expressed recombinant SnuCalCp03-propeptide (rSnuCalCp03-propeptide) showed lowest IC_50_ value of 19 ± 2.4 nM against cathepsin L and the time course indicated the action of a slow-binding reversible inhibitor. Because of the time-dependent formation of the *EI* complex, it is possible to determine the pre-steady state kinetic parameters using the progress curves of product formation. The individual parameters (*v*_i_, *v*_s_, and *k*_obs_) were used to estimate the pre-steady state kinetic parameters *k*_on_ and *k*_off_ , and the dissociation constant *K*_i_ using the Equations (1–5).

(1) $$EI\overset{k_{off}}{\underset{k_{on}[I]}{⇌}}E\overset{K_{m}}{⇄}ES\overset{k_{cat}}{\to }E+P$$

(2) $$[P]=v_{i}*t+\frac{\left(v_{i}-v_{s})[1-\exp^{\left(-k_{obs}*t\right)}\right]}{k_{obs}}$$

(3) $$K_{i}=\frac{\left[I\right]}{v_{i}/v_{s}-1}$$

(4) $$k_{on}=\frac{k_{obs}}{[I]+K_{i}}$$

(5) $$k_{off}=k_{on}*K_{i}$$

Cathepsin propeptides are slow-binding inhibitors of their respective mature enzymes and this inhibition can be described by a single-step mechanism^19^. Two conditions are satisfied for inhibition to fit to an apparent single-step mechanism: *k*_obs_ (the observed pseudo-first-order rate constant) is linearly dependent on the inhibitor concentration and the initial velocity is not affected by inhibitor concentration. The time course in Figure 1(A) showed little or no change in *v*_i_ during the initial stages of the reaction, and a plot of *k*_obs_ vs [*I*] in Figure 1(B) remains linear over the range of inhibitor concentration studied (5.3–42.5 nM), suggesting that inhibition of cathepsin L by rSnuCalCp03-propeptide occurs through a one-step process. The values of *v*_i_, *v*_s_, and *k*_obs_ obtained as described in Equation (2) can be replotted using Equation (3) and the relationship of “ *K*_i_ = [*I*]/(*v*_i_/*v*_s_)-1” to obtain values for the parameters (*k*_on_ and *k*_off_) defined by Equations (4) and (5)^20^ (Figure 1(C)). The values of *K*_i_, *k*_on_, and *k*_off_ were determined as shown in Table 3.

**Table 3.** Kinetic parameters for the inhibition of cathepsin L with propeptides.

Propeptide	*k*_on_ (× 10^6^ M^−1^s^−1^)	*k*_off_ (× 10^−3^s^−1^)	*K*_i_ (nM)
rSnuCalCp03-propeptide	1.08	1.36	2.26
Cathepsin L propeptide (*Homo sapiens*)	12.07	0.99	0.08
CTLA-2β (*Mus musculus*)	–	–	24.00
Rec BCPI (*Bombyx mori*)	–	–	0.11
*Drosophila* CTLA-2-like protein (*Drosophila melanogaster*)	–	–	3.90
Compound **7** tripeptide	–	–	19.00

The low nanomolar *K*_i_ (2.3 ± 0.2 nM) values obtained from this study are comparable to those of the previous studies^21^. Although the *K*_i_ value of rSnuCalCp03-propeptide was 30-fold higher than those of native cathepsin L propeptide and Rec BCPI (*Bombyx mori*), the rSnuCalCp03-propeptide showed a much lower *K*_i_ value than those of CTLA-2β (*Mus musculus*), *Drosophila* CTLA-2-like protein (*Drosophila melanogaster*), and synthetic peptide inhibitors, indicating that it is a relatively potent inhibitor^22–25^. There are some minor differences between the reported values and those determined herein. These can be attributed to the differences in the composition of assay buffers, recombinant cathepsin, and temperature used in the present and previous studies.

A tumor cell invasion assay was accomplished to determine if the *in vitro* inhibition of the cathepsin L-like proteases by rSnuCalCp03-propeptide results in blockage of tumor cell invasion. H1975, HT29, MDA-MB-231, PANC1, and PC3 cell lines were selected due to the high correlation of the cathepsin L-like protease expression level with brain- and lung-specific metastases. The presence of the different cathepsin L-like subfamily proteases within the MDA-MB-231 cell line was verified by reverse transcription PCR. H1975, HT29, MDA-MB-231, PANC1, and PC3 cell lines were used in a series of *in vitro* invasion assays to ascertain the extent to which the secreted cathepsin L-like proteases promote tumor cell invasion through their inhibition by the rSnuCalCp03-propeptide (400 nM) for 24 h. We determined the relative rates of invasion through the Matrigel-coated Transwells. As controls, an unrelated recombinant protein produced from the same bacterial expression vector (400 nM) and a buffer control were also incubated with the cells under identical conditions. The rSnuCalCp03-propeptide was shown to reduce the rate of tumor cell invasion.

Activation of the protease zymogen should occur under conditions where the propeptide no longer inhibits the enzyme. This can be accomplished either by cleavage of the prodomain into noninhibitory fragments, generally by another protease such as in the coagulation cascade, or by the conformational changes of the prodomain in response to the medium changes (e.g. acidic pH, binding of anionic oligosaccharides or membranes) with subsequent cleavage either intra- or intermolecularly. At acidic pH, the weaker inhibition of cathepsin L by free rSnuCalCp03-propeptide is in accordance with an autocatalytic reaction mechanism that is triggered by a drop in pH because of a tight complex that forms between the prodomain and the peptidase domain of proenzyme at pH 6.0. Upon changing to an acidic pH, the proenzyme undergoes a conformational transition and becomes more susceptible to proteolysis. The comparison of the far-ultraviolet CD spectra of rSnuCalCp03-propeptide at pH 4–6 supports the above mentioned hypothesis of zymogen activation and proves its inhibitory activity simultaneously (Figure 3(C)).

## 5. Conclusion

The potent propeptide inhibitor candidates were isolated and characterized from the prodomain of cysteine proteases by comparative structural analysis based on the amino acid sequence and inhibitory activity against human cathepsin L. Among the candidates, cloned and expressed rSnuCalCp03-propeptide out of 19 candidates showed a low nanomolar *K*_i_ value of 2.3 ± 0.2 nM against cathepsin L and a significant inhibition of tumor cell invasion was observed against H1975, HT29, MDA-MB-231, PANC1, and PC3 with a 76, 67, 67, 63, and 79% respective reduction in invasion determined by the rSnuCalCp03-propeptide of 400 mM. Overexpressed recombinant propeptide from *Calotropis procera* R. br. was capable of effectively inhibiting cathepsin L activity for practical applications. Therefore, it is still a better strategy to screen and characterize new inhibitors from natural sources than to synthesize chemical and biological inhibitors by rational or computational design.

## Supplementary Material

IENZ_1444609_Supplementary_Material.pdf
