# Supplementary material for: Molecular cloning and anti-invasive activity of cathepsin L propeptide-like protein from Calotropis procera R. Br. against cancer cells
Source: J Enzyme Inhib Med Chem. 2018 Mar 21;33(1):657–64. doi: 10.1080/14756366.2018.1444609 (PMC6010012; doi:10.1080/14756366.2018.1444609)
Supplement: IENZ_1444609_Supplementary_Material.pdf [file IENZ_A_1444609_SM6301.pdf]

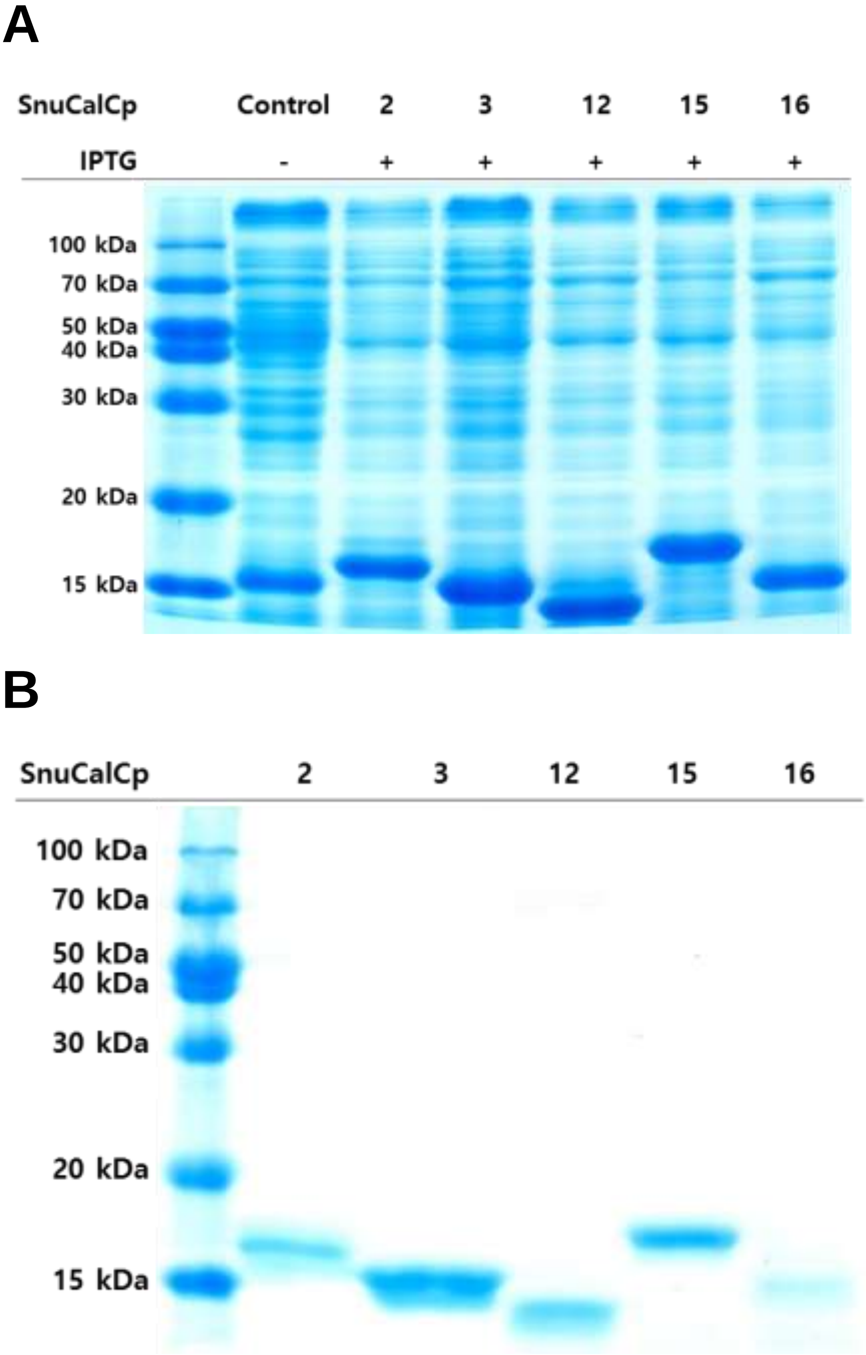

S1 Figure. Expression and purification of rSnuCalCp-propeptide. (A) Expression of the rSnuCalCp-propeptide was induced using IPTG and cell lysates were taken from an uninduced control and induced *Echerichia coli* cultures. SDS-PAGE analysis confirmed expression and inducibility of the rSnuCalCp-propeptide at 13-16 kDa. (B) The rSnuCalCp-propeptide was purified by a series of purification procedures including Ni-NTA affinity, Hitrap Q anion exchange, and HiLoad 16/60 Superdex size exclusion chromatography. The purity of final fractions was analyzed by SDS-PAGE with Coomassie staining.

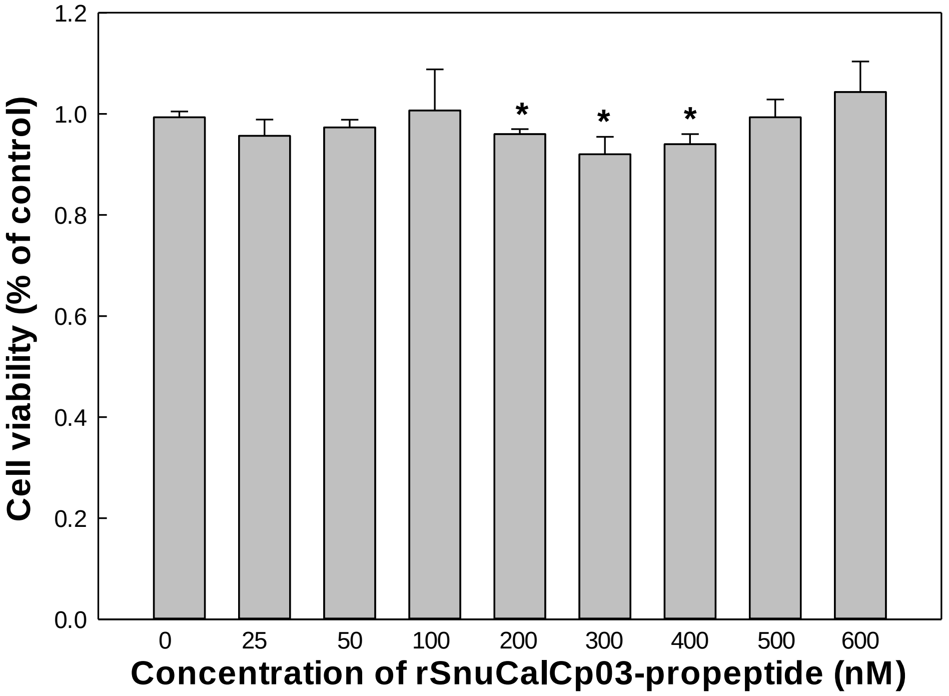

S2 Figure. MTT assay was done on MDA-MB-231 cells and incubated with rSnuCalCp03-propeptide for 24 h. The Student's t test was used to determine the statistical significance of the reduction in invasion observed in the presence of the rSnuCalCp03-propeptide (*, $P < 0.05$).

S1 Table. List of primer sequence for cloning of recombinant cathepsin L propeptide-like protein.

Unigene	Forward primer (5′ to 3′)	Reverse primer (5′ to 3′)
SnuCalCp02	AAGGAGATATACATATGTT AGACATGTCCATTATCAGT	GGTGGTGGTGCTCGAGATCAT CACCAGCATTAAAAGA
SnuCalCp03	AAGGAGATATACATATGAA AATCATATCCATTGCCGAT	GGTGGTGGTGCTCGAGGTTAA GGTCAACTTCAGAAAA
SnuCalCp08	AAGGAGATATACATATGGTT GACGACGGATCATCAG	GGTGGTGGTGCTCGAGGACAA CGTTGGTTAGCTTG
SnuCalCp12	AAGGAGATATACATATGAT TGCCGATGAATTAGTCCG	GGTGGTGGTGCTCGAGAGAAA GGTTATAGTTAACTTGG
SnuCalCp14	AAGGAGATATACATATGTC ATTTTCATCTTCTTCTTCTT	GGTGGTGGTGCTCGAGATCAA AATCATCAAAGACATCT
SnuCalCp15	AAGGAGATATACATATGAT CATCACTACTAGCCTCC	GGTGGTGGTGCTCGAGGCTTT CAGATCCAACTTTGT
SnuCalCp16	AAGGAGATATACATATGGA CCGTTCATCATTCTCCG	GGTGGTGGTGCTCGAGAGCAA CGTTGTTGAGCTTTA
SnuCalCp17	AAGGAGATATACATATGTCT GAGATCACGTCGGTTA	GGTGGTGGTGCTCGAGATTATC TTCGGCTTTAGGAAG
